# GO2PUB: Querying PubMed with semantic expansion of gene ontology terms

**DOI:** 10.1186/2041-1480-3-7

**Published:** 2012-09-07

**Authors:** Charles Bettembourg, Christian Diot, Anita Burgun, Olivier Dameron

**Affiliations:** 1 UMR936, INSERM, Université de Rennes 1, 2 av. Léon Bernard, F-35043 Rennes, France; 2 UMR1348, INRA, Agrocampus Ouest, 65 rue de Saint-Brieuc, F-35042 Rennes, France

**Keywords:** Gene ontology, Semantic expansion, Query enrichment, PubMed

## Abstract

**Background:**

With the development of high throughput methods of gene analyses, there is a growing need for mining tools to retrieve relevant articles in PubMed. As PubMed grows, literature searches become more complex and time-consuming. Automated search tools with good precision and recall are necessary. We developed GO2PUB to automatically enrich PubMed queries with gene names, symbols and synonyms annotated by a GO term of interest or one of its descendants.

**Results:**

GO2PUB enriches PubMed queries based on selected GO terms and keywords. It processes the result and displays the PMID, title, authors, abstract and bibliographic references of the articles. Gene names, symbols and synonyms that have been generated as extra keywords from the GO terms are also highlighted. GO2PUB is based on a semantic expansion of PubMed queries using the semantic inheritance between terms through the GO graph. Two experts manually assessed the relevance of GO2PUB, GoPubMed and PubMed on three queries about lipid metabolism. Experts’ agreement was high (kappa = 0.88). GO2PUB returned 69% of the relevant articles, GoPubMed: 40% and PubMed: 29%. GO2PUB and GoPubMed have 17% of their results in common, corresponding to 24% of the total number of relevant results. 70% of the articles returned by more than one tool were relevant. 36% of the relevant articles were returned only by GO2PUB, 17% only by GoPubMed and 14% only by PubMed. For determining whether these results can be generalized, we generated twenty queries based on random GO terms with a granularity similar to those of the first three queries and compared the proportions of GO2PUB and GoPubMed results. These were respectively of 77% and 40% for the first queries, and of 70% and 38% for the random queries. The two experts also assessed the relevance of seven of the twenty queries (the three related to lipid metabolism and four related to other domains). Expert agreement was high (0.93 and 0.8). GO2PUB and GoPubMed performances were similar to those of the first queries.

**Conclusions:**

We demonstrated that the use of genes annotated by either GO terms of interest or a descendant of these GO terms yields some relevant articles ignored by other tools. The comparison of GO2PUB, based on semantic expansion, with GoPubMed, based on text mining techniques, showed that both tools are complementary. The analysis of the randomly-generated queries suggests that the results obtained about lipid metabolism can be generalized to other biological processes. GO2PUB is available at http://go2pub.genouest.org.

## Background

The development of high-throughput methods of gene analysis requires to deal with lists of thousands of genes while researchers were used to search the literature only for a few genes at a time. The information retrieval process becomes an increasingly difficult task and needs to be redesigned to provide literature concerning biological problems raised by the gene analyses.

PubMed is the most comprehensive public database of biomedical literature. It comprises more than 21 million entries for biomedical literature from MEDLINE, life science journals, and online books^a^. The typical PubMed user has to read several dozens to hundreds of abstracts to select the relevant ones. More than 4 million articles were added in the last 5 years ^b^.

A well defined query is important to retrieve as many relevant articles as possible with as few irrelevant ones as possible. Such a query is often more complex than the few loosely-coupled keywords used by most users. There is a need for automatic tools helping the users to build such complex queries that minimize silence and noise [1,2].

Although PubMed supports MeSH-based query expansion [3], other literature search tools have been developed [4-7] and evaluated [8]. These can be classified into three major approaches. The first approach, exemplified by tools like SLIM [9], is based on an intuitive interface to set some filters on PubMed queries in order to obtain a better precision than with the basic PubMed querying system. A good proficiency with PubMed *advanced search* brings similar results.

The second approach developed in SEGOPubMed uses a Latent Semantic Analysis (LSA) framework. It is based on a semantic similarity measure between the user query and PubMed abstracts [10]. The authors of SEGOPubMed state that the LSA approach outperforms the other approaches when using well-referenced keywords. Unfortunately, no implementation of SEGOPubMed is currently available. Moreover, this method requires that a corpus of well-referenced keywords be constituted and maintained before the search. Such a corpus is not available (in the biomedical domain) either.

The third approach is based on query enrichment using controlled vocabularies and ontologies. An ontology is a knowledge representation in which concepts are described both by their meaning and their relations to each other [11]. Ontologies are useful to find information relevant to a given topic, particularly through a query expansion process[12]. The automatic handling of the query complexity facilitates query formulation. Expanded queries applied to the web information retrieval show a systematic improvement over the unexpanded ones [13]. QuExT performs a concept-oriented query expansion to retrieve articles associated with a given list of genes symbols from PubMed and to prioritize them [14].

However, a frequent goal of gene-related analyses (e.g. transcriptomics) is to identify the genes with different expression across samples analyzed. Thereafter, scientists link their list of genes to more synthetic keywords and functions using Gene Ontology (GO) terms [15] associated to genes thanks to the Gene Ontology Annotation database [16]. At this stage of the gene-related analyses, the keywords to search the literature are not gene names anymore but GO terms. Therefore, tools querying litterature with GO terms seem appropriate. GoPubMed [17] uses a text extraction algorithm to mine PubMed abstracts with GO terms. It relies on a local string alignment to compare the GO terms and the abstracts. GoPubMed selects the abstracts containing at least a significant part of the semantic of the GO terms. However, GoPubMed does not follow GO strict rules conveying the semantics of terms. If the annotation of a gene product gp by a Gene Ontology term t is true, then the annotation of gp by any parent of t is equally true [16]. All transitive relation (is a, part of) have to be followed to retrieve these parents. As GoPubMed does not follow this rule, its recall decreases whenever inferences about gene annotations yield new relevant results [18]. None of the existing tools supports a combination of semantics-based and of synonym-based PubMed query enrichment.

In this study, we hypothesized that the name of the genes annotated by a GO term of interest or one of its descendants can be used as keyword in gene-oriented PubMed queries. The descendants of a GO term are defined according to the Gene Ontology specifications of reasoning about relations^c^. The genes annotated with GO terms are provided by the Gene Ontology Annotation database.

In our system GO2PUB, we propose a new approach that considers not only the genes annotated with a GO term of interest, but also those annotated by a descendant of this GO term, complying with the semantic inheritance properties of GO. GO2PUB’s user inputs a list of GO terms of interest, one or more species, and a list of keywords. It generates a PubMed query with the names, symbols and synonyms or aliases of these genes, the species and the keywords and processes PubMed results.

We performed a qualitative relevance study on our domain of expertise using three queries related to lipid metabolism. Because GO2PUB and GoPubMed both use GO terms as input we wanted to confront the results from these tools. For each query, we compared GO2PUB results with those of the original GoPubMed and of GoPubMed after having manually-generated the semantic expansion of the GO terms. In addition, we submitted similar queries to PubMed as it is the reference literature search tool. Two experts manually determined the relevance of all the articles. We computed the precision, relative recall and F-score of GO2PUB, GoPubMed and PubMed. In order to determine if the results of the qualitative study could be generalized, we then performed a study on twenty randomly-generated queries. This study focused on the number of common results and tool-specific results. We also analyzed the relevance of seven of these twenty random queries.

## Results

### Qualitative study

In order to evaluate GO2PUB’s relevance and to compare it with GoPubMed, we assessed three queries (Q1, Q2 and Q3) about biological processes related to lipid metabolism and including different GO terms, species and MeSH terms. We submitted our queries to GoPubMed using the same keywords and tags. As GoPubMed only considers the GO term(s) provided by the user and ignores the inheritance rules of Gene Ontology, we also expanded queries manually then submitted them to GoPubMed. Our GoPubMed queries were composed of the GO term(s) and all its descendants separated by “OR”, plus MeSH keywords. This ensured the closest comparison possible. We also constructed the PubMed queries as close as possible to our GO2PUB queries.

#### Relevance criteria

The role of GO2PUB is to retrieve literature about gene functions summarized by a GO term thanks to a gene analysis process. We analyzed the results of GO2PUB, GoPubMed and PubMed queries according to the following criteria. We considered that a relevant article had to describe at least one gene product occurring in the chosen domain of interest for the selected species. The gene product’s description has to focus on its role, its interactions, and how and when it is activated.

For each query Q1, Q2 and Q3, the results obtained by the different tools were mixed for a blind selection by a biologist, CD and by a bioinformatician, CB. This ensured that the reviewers did not know which tool(s) retrieved the articles. The final list of relevant articles is the union of the two reviewers’ lists.

#### Relevance measurement

For each query and tool, we computed the precision, recall and F-score. Computing the recall for each query is impossible because it would require to know all the relevant articles available in Medline. As it is possible that some of these articles were missed by all three tools, recall was defined as relative to all relevant articles obtained by at least one of the tools.

**Figure 1:**
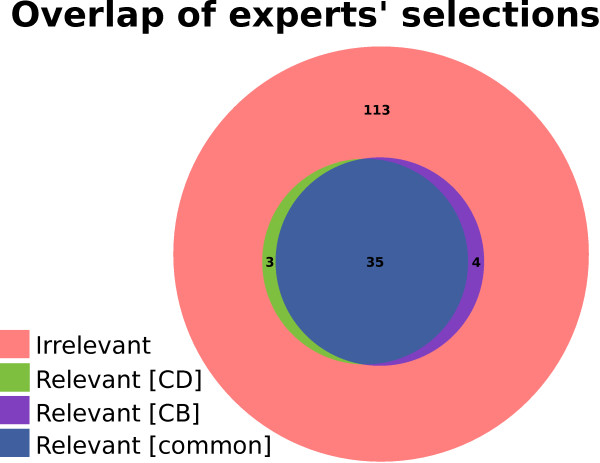
**Comparison of the experts’ relevance selections.** Experts’ selections overlap of relevant articles among all obtained as results from the three reviewed queries (Q1, Q2 and Q3).

Figure 1 presents the reviewers’ selections of relevant articles among all the results of the qualitative study. Most of the relevant articles were found in the intersection of the two selections. Reviewers agreed on 35 relevant and 113 irrelevant articles while selecting separately 3 and 4 articles as relevant. Additional files 1, 2 and 3 provide the experts’ selections for Q1, Q2 and Q3.

We used Cohen’s kappa coefficient as a statistical measure of inter-rater agreement [19]. The value obtained was 0.88, which corresponds to an almost perfect agreement [20].

#### Query Q1: Lipogenesis in chicken liver

For our first query in GO2PUB, we used “Lipid biosynthetic process” (GO:0008610) as GO term, “Gallus gallus” as species, “Liver” as Major Topic and “Lipid Metabolism” as MeSH keyword, and we considered the articles published in the last five years. We ran query Q1 on GO2PUB using the [BASICq], [MeSHq] and [ORq] options described in method section. “Lipid biosynthetic process” has 243 descendants in the GO graph. The mean number of edges to reach the root of the ontology from this term was 3.5.

Additional file 4 contents the results obtained by GO2PUB for query Q1. Results are formatted for a quick access to information. Each citation obtained from PubMed is listed; the title, authors, date, abstract, journal, PMID and MeSH terms are displayed. The name, symbol and synonyms of gene annotated by the GO term(s) are highlighted in the title and abstract.

The query Q1 formulated for GoPubMed included *“lipid biosynthetic process”[go] AND Chickens[mesh] AND Liver[majr] AND “Lipid Metabolism”[mesh] AND last5years[time]*. This is the “standard” query for GoPubMed. We also formed the manually-expanded version of this query by adding the descendants of “lipid biosynthetic process” separated by “OR”. It should be noted that 47 of the 243 terms generated by the semantic expansion of “Lipid biosynthetic process” generated a GoPubMed error and had to be ignored. For example, one of the descendants of “Lipid biosynthetic process” is “Regulation of phospholipid biosynthetic process” (GO:0071071), which is a relevant descendant term. When querying GoPubMed with this GO term, we obtained an error: “Your query could not be understood: Can’t find a term regulation of phospholipid biosynthetic process”.

The PubMed equivalent query for Q1 was “Chickens liver lipogenesis”, which PubMed interpreted as *(“chickens”[mesh] OR “chickens”[all]) AND (“liver”[mesh] OR “liver”[all]) AND (“lipogenesis”[mesh] OR “lipogenesis”[all])*.

**Figure 2:**
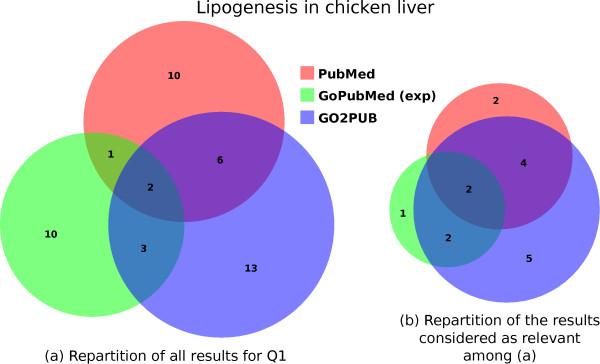
**Comparison of the PubMed, GoPubMed and GO2PUB results for query Q1.** (**a**) displays the repartition and intersections of these results. (**b**) displays the repartition and intersections of the results considered as relevant.

Figure 2 presents Venn diagrams comparing the results obtained with PubMed, GoPubMed (after manual expansion) and GO2PUB for Q1. Figure 2A presents the raw results. Although queries as similar as possible were issued to the three tools, the resulting sets of articles had little overlap. Figure 2B presents the repartition of the relevant articles. Most of the relevant articles were identified by GO2PUB. Of note, most of the articles retrieved by at least two tools (overlaps in Figure 2A) were found to be relevant (overlaps in Figure 2B).

Table 1 presents the precision, relative recall and F-score for each tool. GO2PUB had a better precision and relative recall than GoPubMed and Pubmed. Regarding GoPubMed, there was no difference between the “standard” and “expanded” results.

**Table 1:** Measures for query Q1

	**PubMed**	**GoPM (std)**	**GoPM (exp)**	**GO2PUB**
(a) Number of results	19	16	16	24
(b) Relevant among (a)	8	5	5	13
Precision	0.421	0.313	0.313	0.542
Relative Recall	0.5	0.313	0.313	0.813
F-score	0.457	0.313	0.313	0.650

Values of precision, relative recall and F-score using PubMed, GoPubMed without (GoPM std) or with (GoPM exp) manual expansion and GO2PUB search tools for query Q1 about lipogenesis in chicken liver. Values are calculated from (a) and (b) lines using a total number of relevant results of 16.

#### Query Q2: Lipid transport in human blood

In our second reviewed query in GO2PUB, we used “Lipid transport” (GO:0006869) as GO term, “Homo sapiens” as species, “Blood” as Major Topic and “Lipid Metabolism” as MeSH keyword, and we considered the articles published in the last five years. “Lipid transport” has 109 descendants in the GO graph. The mean number of edges to reach the root of the ontology from this term was 4.3.

We ran equivalent queries on GoPubMed (“standard” and “expanded” versions) and PubMed. 46 of the 109 terms generated by the semantic expansion of “Lipid Transport” generated a GoPubMed error and had to be ignored.

As there is no MeSH term for “lipid transport”, we searched it in titles and abstracts on PubMed. The PubMed query was: *“lipid transport”[TIAB] AND ((“blood”[Subheading] OR “blood”[All Fields] OR “blood”[MeSH Terms]) AND (“humans”[MeSH Terms] OR “humans”[All Fields] OR “human”[All Fields])) AND (“2006/03/28”[PDat] : “2011/03/28”[PDat])*.

**Figure 3:**
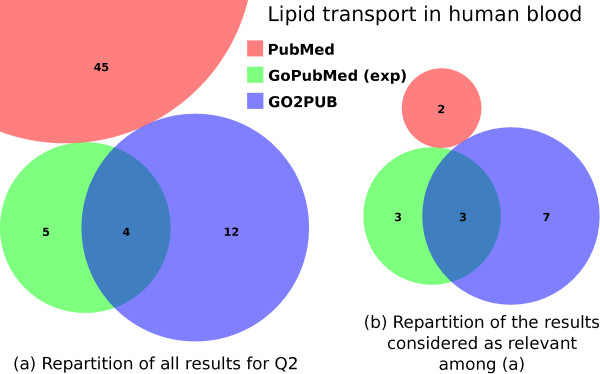
**Comparison of the PubMed, GoPubMed and GO2PUB results for query Q2.** (**a**) displays the repartition and intersections of these results. (**b**) displays the repartition and intersections of the results considered as relevant.

Figure 3 presents the results obtained by PubMed, GoPubMed (after manual expansion) and GO2PUB for Q2. As observed for query Q1, the majority of the results were tool-specific. PubMed yielded 45 articles, none of which were retrieved by GO2PUB nor GoPubMed while there was an overlap between GO2PUB and GoPubMed results. Considering only GoPubMed and GO2PUB, most of the results were specific to one tool or the other and few were obtained by both tools (Figure 3A). Three of the four common articles between GoPubMed and GO2PUB were relevant (Figure 3B). GO2PUB yielded half of GoPubMed relevant results while having an important specific relevant results set. Only 2 article on 45 yielded by PubMed were relevant.

Table 2 presents precision, relative recall and F-score for each tool. GO2PUB has a slightly lower precision than GoPubMed (standard and after manual expansion) but better relative recall and F-score. For GoPubMed, there was no difference between “standard” and “expanded” results.

**Table 2:** Measures for query Q2

	**PubMed**	**GoPM (std)**	**GoPM (exp)**	**GO2PUB**
(a) Number of results	45	9	9	16
(b) Relevant among (a)	2	6	6	10
Precision	0.044	0.667	0.667	0.625
Relative Recall	0.133	0.4	0.4	0.667
F-score	0.067	0.5	0.5	0.645

Values of precision, relative recall and F-score using GoPubMed without (GoPM std) or with (GoPM exp) manual expansion and GO2PUB search tools for query Q2 about lipid transport in human blood. Values are calculated from (a) and (b) lines using a total number of relevant results of 15.

#### Query Q3: Regulation of lipase activity in human cell membrane

Our third query in GO2PUB used “Regulation of lipase activity” (GO:0060191) as GO term, “Homo sapiens” as species and “Cell Membrane” and “Lipid Metabolism” as MeSH keywords, and considered the articles published in the last ten years. “Regulation of lipase activity” has 35 descendants in the GO graph. The mean number of edges to reach the root of the ontology from this term was 5.25.

We ran equivalent queries on GoPubMed (“standard” and “expanded” versions) and PubMed. 16 of the 35 terms generated by the semantic expansion of “Regulation of lipase activity” generated a GoPubMed error and had to be ignored.

The PubMed query was composed of the keywords “regulation”, (“lipase” AND “activity”), “human” and (“cell” AND “membrane”).

**Figure 4:**
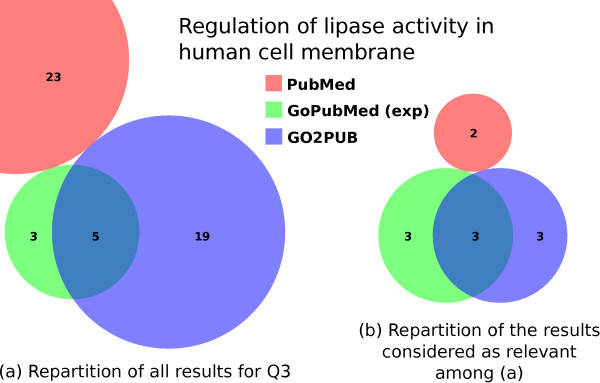
**Comparison of the PubMed, GoPubMed and GO2PUB results for query Q3.** (**a**) displays the repartition and intersections of these results. (**b**) displays the repartition and intersections of the results considered as relevant. The GoPubMed displayed set is the one that uses the manual semantic expansion.

Figure 4 presents the results obtained by PubMed, GoPubMed (after manual expansion) and GO2PUB. Figure 4A shows a larger set of results for GO2PUB compared to GoPubMed (24 and 8, respectively), but we can see in Figure 4B that most of these results are irrelevant. As observed for query Q2, none of the PubMed results were retrieved by GO2PUB nor GoPubMed while there was an overlap between GO2PUB and GoPubMed results. Only 2 articles on 23 identified by PubMed were relevant.

Table 3 presents precision, relative recall and F-score for each tool. GO2PUB has a relative recall equivalent to GoPubMed’s and a lower precision and F-score. For GoPubMed, the “manually-expanded” results have a higher relative recall and F-score and a lower precision than the “standard” ones. We observed again a discrepancy between PubMed and the other tools, with a lower precision, a lower relative recall, and consequently a lower F-score for PubMed.

**Table 3:** Measures for query Q3

	**PubMed**	**GoPM (std)**	**GoPM (exp)**	**GO2PUB**
(a) Number of results	23	6	8	24
(b) Relevant among (a)	2	5	6	6
Precision	0.087	0.833	0.75	0.25
Relative Recall	0.182	0.455	0.545	0.545
F-score	0.118	0.588	0.632	0.343

Values of precision, relative recall and F-score using PubMed, GoPubMed without (GoPM std) or with (GoPM exp) manual expansion and GO2PUB search tools for query Q3 about regulation of lipase activity in human cell membrane. Values are calculated from (a) and (b) lines using a total number of relevant results of 11.

### Generalization study

In order to determine whether previous results are representative of GO2PUB’s performances, we performed a generalization study on twenty randomly-generated queries. We compared the profile of the results obtained by GO2PUB and GoPubMed in this generalization study with those obtained in the qualitative study. This profile depends on the average size of the sets of articles. The following proportions were calculated on the result set constituted by all GoPubMed and GO2PUB results. In the qualitative study, GO2PUB yielded 21.33 articles on average, which represented 77.1% of the total. GoPubMed yielded 11.0 articles on average, which represented 39.8% of the total. There were 4.67 articles on average in the set of common articles, which represented 16.9% of the total.

We built queries following the pattern: “a random GO term + a species (mouse) + a publication date limit (2011) + a keyword (the GO term name)”. To be coherent with our qualitative study, we randomly selected twenty GO terms among all Biological Process terms having a granularity similar to those of the three GO terms used in the qualitative study. We assumed that the granularity of a term depends on the mean length of its path to the root, and its number of descendants. Each GO term of the generalization study had a mean path length to the root between 3.5 and 5.25 edges and had between 35 and 244 descendants. As we could not add a MeSH keyword in relation with the random GO term of each query, we simply added the name of this GO term. This keyword was added in the free field for GO2PUB and without [go] tag for GoPubMed. We submitted these queries to GO2PUB and to GoPubMed.

**Figure 5:**
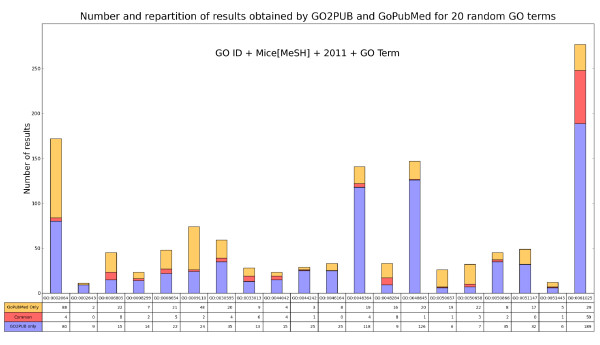
**Repartition of results obtained by GO2PUB and GoPubMed for twenty general GO queries without free keyword.** This diagram presents the sets of results obtained by GoPubMed and GO2PUB for twenty general queries. These queries were built with a random GO term having a granularity similar to that of GO terms of the qualitative study.

Figure 5 presents the sets of articles obtained by GO2PUB and GoPubMed for these queries. GO2PUB yielded 46 articles on average (min 6, max 189) compared to 21.33 on the qualitative study. They represented 70.4% of the total number of articles (77.1% in the qualitative study). GoPubMed yielded 25.1 articles on average (min 2, max 88) compared to 11.0 on the qualitative study. They represented 38.4% of the total number of articles (39.8% in the qualitative study). There were 5.75 articles on average (min 0, max 59) in the common set. They represented 8.8% of the total (16.9% in the qualitative study). The profile of these results is close to the qualitative study one.

We studied the relevance of the results from seven queries picked out among the twenty queries of the generalization study. Out of the seven queries, three were chosen because they were in our reviewers’ domain of expertise: “cellular lipid catabolic process” [GO:0044242], “isoprenoid biosynthetic process” [GO:0008299] and “phospholipid biosynthetic process”[GO:0008654]. Cohen’s kappa was 0.9345. We picked randomly four additional queries about “RNA transport” [GO:0050658], “tetrapyrrole metabolic process” [GO:0033013], “xenobiotic metabolic process” [GO:0006805] and “organelle fusion” [GO:0048284]. Cohen’s kappa remained high for these four queries (0.797) in spite of them being out of our reviewers’ domain of expertise. Table 4 presents the number of results, precision, relative recall and F-score respectively for the three lipid-related queries and the other four queries of the generalization study. Results are similar to those observed in the qualitative study. The resulting sets of articles had little overlap. Moreover, each tool yielded relevant results ignored by the other, with important variation of performances among queries.

**Table 4:** Measures for seven generalization queries

**(A) Lipids**	**GO:0044242**	**GO:0008299**	**GO:0008654**					
Tool	GPM	G2P	GPM	G2P	GPM	G2P		
(a) Number of results	4	26	9	16	25	27		
(b) Relevant among (a)	3	20	1	2	5	11		
(c) Total relevant	22	3	12					
(d) Common results	1	2	5					
(e) Relevant among (d)	1	0	4					
Precision	0.750	0.769	0.111	0.125	0.200	0.407		
Relative Recall	0.136	0.864	0.333	0.667	0.417	0.917		
F-score	0.231	0.814	0.167	0.211	0.270	0.564		
**(B) Other**	**GO:0050658**	**GO:0033013**	**GO:0006805**	**GO:0048284**				
Tool	GPM	G2P	GPM	G2P	GPM	G2P	GPM	G2P
(a) Number of results	25	10	15	19	30	23	24	17
(b) Relevant among (a)	7	2	3	3	17	14	10	9
(c) Total relevant	9	6	26	16				
(d) Common results	3	6	7	8				
(e) Relevant among (d)	1	2	4	4				
Precision	0.280	0.200	0.200	0.158	0.567	0.609	0.417	0.529
Relative Recall	0.875	0.250	0.600	0.600	0.680	0.560	0.625	0.563
F-score	0.424	0.222	0.300	0.250	0.618	0.583	0.500	0.545

Results’ sets sizes and values of precision, relative recall and F-score using GoPubMed and GO2PUB search tools for seven random queries: three lipid-related queries (part A) and four queries about other topics (part B). Values of precision, relative recall and F-score are calculated from (a), (b) and (c) lines. (d) and (e) lines provide GO2PUB and GoPubMed common results’ sets sizes and the number of common relevant results.

## Discussion

Our goal was to develop a tool that uses the knowledge from the Gene Ontology (GO) and its annotations for generating semantically-expanded gene-related PubMed queries. Indeed, there is no [GO] tag for a search in PubMed.

The qualitative study showed that both GO2PUB and GoPubMed retrieved relevant articles ignored by PubMed. For the query Q1 about lipogenesis in chicken liver, 26 of the 35 articles (8 of 14 relevant) returned by either GO2PUB or GoPubMed were ignored by PubMed. Conversely, 9 of the 19 articles (6 out of 8 relevant) returned by PubMed were also returned by either GO2PUB or GoPubMed. For Q2 and Q3, the set of articles returned by PubMed was disjoint from both GO2PUB and GoPubMed results. PubMed identified only 4 relevant articles not yielded by GO2PUB nor GoPubMed for these 2 queries.

Overall, GO2PUB performed better than GoPubMed and PubMed. Both GoPubMed and GO2PUB and to a lesser extend PubMed yielded relevant articles ignored by the others. The discrepancy observed between PubMed and the other tools is probably due to the absence of a [GO] search field tag in PubMed. GO2PUB performance varied among the queries. For two queries (Q1, Q2) of the qualitative study, GO2PUB yielded most of the relevant articles and had therefore the highest relative recall value while its precision was slightly lower than that of GoPubMed. Consequently, GO2PUB had the best F-score. For Q3, GO2PUB yielded as many relevant articles as GoPubMed but had a higher noise proportion. GO2PUB had a slightly better relative recall than GoPubMed, but its precision was much lower. Consequently, GoPubMed had the best F-score. We can also notice that for Q3, the query expansion on GoPubMed improved its performances with a better relative recall and F-score at the cost of a small loss of precision. We observed similar results on the seven queries of the generalization study for which we assessed the relevance.

GO2PUB performs a semantic expansion of the GO terms of interest complying with the semantic inheritance through the GO graph before retrieving the corresponding genes to enrich the query. All the results of GO2PUB presented here were obtained using the concept of query expansion. During the development of GO2PUB, we also ran queries without this expansion. We obtained empty or very small sets of results.

Using the semantic inheritance properties of the GO graph is useful. The more descendants a GO term has, the more relevant results GO2PUB yields. GO2PUB performance decreased from Q1 to Q3. For Q1, “lipid biosynthetic process” has 243 descendants and annotates 646 genes for human and 145 genes for chicken. For Q2, “lipid transport” has 109 descendants and annotates 253 genes for human and 63 genes for chicken. For Q3, “regulation of lipase activity” has 35 descendants and annotates 168 genes for human and 18 genes for chicken. The more descendants a GO term has, the more genes it is likely to annotate. Moreover, Q1 concerned chicken, which is less annotated than human. On less annotated species, the annotations focus on the major genes. This explains why GO2PUB yields a high proportion of relevant articles.

Concerning Q3, GO2PUB had a low precision compared to GoPubMed. Genes annotated with GO terms on regulation usually have many additional functions. Consequently, the articles about genes annotated by “regulation of lipase activity” searched in Q3 may also describe the other functions of these genes. To obtain a better precision in this case, we suggest to further specify the query with a MeSH term or a free keyword.

GoPubMed does not follow the semantic inheritance properties of GO. We manually expanded GoPubMed queries and compared it to GO2PUB. The added value of semantic expansion was null for Q1 and Q2, and important for Q3 (+33%). So query expansion is a built-in functionality in GO2PUB, and would be a valuable extension for GoPubMed. In GoPubMed results, a “missing term” error occurred for 19% of the expanded set of GO terms for Q1, 42% for Q2 and 44% for Q3. We assume that the benefits of query expansion on GoPubMed might be higher when considering the articles related to these currently omitted GO terms.

In order to verify whether the results from the qualitative study on lipid metabolism could be generalized to other domains, we submitted twenty randomly-generated queries to GO2PUB and GoPubMed. Each query contained a random GO term of a granularity similar to that of the terms used in the qualitative study. The proportion of articles returned by GO2PUB was 70.4%, the one of GoPubMed was 38.4% and the proportion of articles returned by both was 8.8%. These proportions were respectively 77.1%, 39.8% and 16.9% for the qualitative study. We assume that the difference of proportions between the qualitative and the generalization studies can be attributed to Q1, Q2 and Q3 being more specific because of the use of MeSH keywords. The seven queries of the generalization study presented relevances similar to those observed in the qualitative study.

GO2PUB seems less suited for queries involving either general GO terms or GO terms with few or no descendants. Indeed, with general GO terms, GO2PUB considers a lot of descendants, and therefore a lot of genes. We expect this to increase the noise as some of the genes will be irrelevant. Conversely, GO terms having few or no descendants are associated with few genes. We do not expect semantic expansion to benefit these highly specific queries yielding only a few PubMed results.

As most of the results obtained by GO2PUB and GoPubMed are relevant in the qualitative study and in the generalization study, the intersection of GoPubMed and GO2PUB results decreases noise. As each tool yields relevant articles ignored by the other, the union of their results also decreases silence.

## Conclusion

GO2PUB brings relevant results ignored by GoPubMed (9 GO2PUB’ specific results for Q1, 7 for Q2 and 3 for Q3) even when adding a manual query expansion for GoPubMed. Conversely GoPubMed text mining approach finds relevant articles ignored by GO2PUB (1 GoPubMed’ specific result for Q1, 3 for Q2 and 3 for Q3). This demonstrates GO2PUB relevance and its complementarity with GoPubMed for our domain of interest. The generalization analysis shows that a similar profile of results is obtained using random queries, especially when using keywords for narrowing the queries. This suggests that the results of the qualitative study can be generalized.

## Resources and methods

### Resources

The files from GO^d^ and GOA^e^ used in our study were downloaded in March 2011. We used the “term” and “term2term” tables of GO for the automatic semantic expansion of GO2PUB and the manual expansion of GoPubMed queries. We used species specific GOA tables to retrieve for each species of interest the gene names annotated by the provided GO terms. These tables allowed us to build queries about seven different species^f^. Since June 2011, GO2PUB uses the Uniprot-GOA table instead of the species-specific tables, allowing researchers to mine the literature about more than 2000 different species. Additionally, this table is more complete than the species-specific tables used previously.

All the queries were submitted to GO2PUB, PubMed and GoPubMed on 28th March 2011. Synonyms and aliases of genes used in GO2PUB were provided by the current version of EntrezGene.

We represented the overlap of the different tools results using Venn diagrams generated by BioVenn [21].

### Methods

#### GO2PUB query building

**Figure 6:**
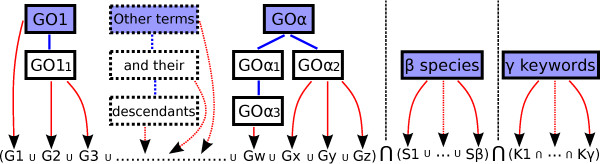
**GO2PUB Query composition process using the parameters provided by the user.** (1) the initial *α*GO terms (purple boxes) are enriched by their descendants. (2) the genes (here noted G1 to Gz) annotated by the GO terms are retrieved. (3) the query is composed using the names, symbols and synonyms of the genes, the *β*species (S) and the *γ*MeSH or free keywords (K).

GO2PUB creates an expanded PubMed query with the name, symbol and synonyms of genes annotated by one or several GO terms provided by the users, for one or several species. Figure 6 presents the process. The users provide one or several GO terms and species. To further restrict their query, they can also provide as many MeSH terms keywords as wanted. Furthermore, a “free text” field supports the use of all the other PubMed tags, like [Author], [Journal], etc., and keywords from MeSH terms or free text.

The first part of each query involves one or more GO terms. The users can enter either the name or the identifier of the GO terms. These terms are suggested when the users start to fill the field. The exact GO term is suggested if the users provide one of its GO synonyms. For example, GO2PUB will search for “lipid biosynthetic process” if the users provide “lipogenesis”. When two or more GO terms are entered, GO2PUB makes the union of them (“OR” connector).

Then, the users select one or several species using a name (common or scientific names and their synonyms are allowed) or a NCBI taxon code^g^. In this case, the users can choose to join them (using “OR”) or intersect them (using “AND”). Logical connectors “AND” and “OR” are set by default to make the union of species and intersection of keywords, but this can be modified.

Next, the users can enter additional MeSH terms to specify their query. MeSH terms associated to the articles by PubMed are not all of same importance, some of them being classified as “Major topic” (MAJR). We can qualify each keyword as a simple MeSH term or a Major topic. Again, the users can specify the connector between keywords.

At this point, the users have built a simple GO2PUB query. We call this query [BASICq]. The system supports three modifications for [BASICq] for studying if minor changes bring additional relevant results.

The first modification ignores MAJR qualifiers and searches all keywords in PubMed [MeSH] tag. As MAJR terms are also MeSH terms, articles associated to them will still be found. We call this query [MeSHq].

The second modification replaces “AND” connectors between keywords by “OR” connectors. However, as it can return substantially more results with a lot of noise, all keywords in this additional query are tagged with MAJR. Species, normally searched in MeSH, are also tagged with MAJR. We call this query [ORq].

The third modification ignores MeSH and MAJR keywords, and tags species with MAJR. This option must be used carefully because it can yield several hundreds of results if the search topic is too large. It is of interest only for very narrow topics if the users do not obtain enough results with the other types of queries. We call this query [NOKq].

Last, GO2PUB proposes three additional options.

The first option sets limits on the publication year.

The second option proposes an exhaustive search of the official synonyms of gene names. It searches Entrez gene^h^ for all the known synonyms for a gene. Since authors sometimes use synonyms that are absent in the GOA database in their articles, this option allows the users to build more complete PubMed queries in order to obtain more relevant results.

The third option toggles the display of the MeSH table associated with each article.

#### Query rewriting using semantic expansion

Semantic expansion consists in following the semantic inheritance through the GO graph in order to also consider all the descendants of the GO terms specified by the users. Then, the process retrieves the gene names annotated with these terms.

**Figure 7:**
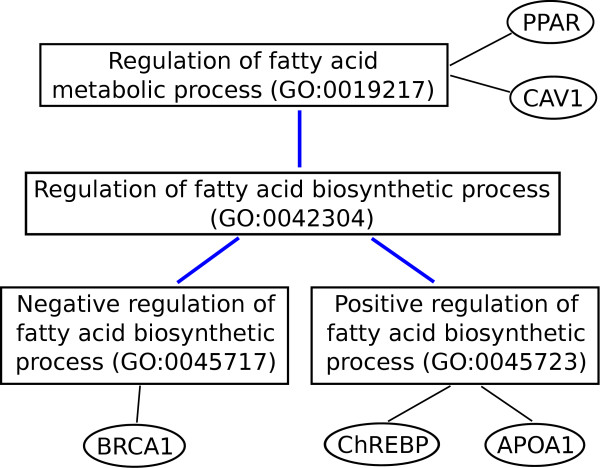
**Keyword semantic enrichment.** For a literature search about the regulation of fatty acid metabolic process, we want to enrich the query with the associated genes. The two genes PPAR and CAV1 are directly annotated by the GO term “Regulation of fatty acid metabolic process” (GO:0019217). However, Gene Ontology inheritance properties say that every term inherits the meaning of all its ancestors. Consequently, genes annotated by at least one descendant of the original term (BRCA1, ChREBP and APOA1) should also be considered.

GO2PUB uses these gene names and their synonyms as additional keywords for PubMed queries. Figure 7 shows that the expansion identifies five genes associated with the regulation of fatty acid metabolic process, instead of two if the semantic inheritance is ignored.

GO2PUB retrieves all gene names annotated by each GO term, directly or indirectly through the semantic inheritance properties. It then builds a query on the model “(*n* gene names, symbols or synonyms separated by OR) AND (*m* species) AND (*p* MeSH terms)”. The name, symbol and synonyms of each gene compose the first part of the query. They will be searched in title and abstract. Species and keywords chosen by the users make up the second part of the query. Finally, GO2PUB submits to PubMed a query composed of gene names annotated directly or indirectly by the GO terms chosen by the users (name OR symbol OR Synonym), at least one species and some MeSH terms and free keywords. This big query is split into several smaller ones if it exceeds PubMed server URL length limitation. GO2PUB compiles the results and displays all citations numbered and sorted by date.

## Endnotes

^a^http://www.ncbi.nlm.nih.gov/pubmed

^b^http://www.nlm.nih.gov/bsd/licensee/baselinestats.html

^c^http://www.geneontology.org/GO.ontology.relations.shtml

^d^go\_daily-termdb-tables.tar.gz from http://archive.geneontology.org/latest-termdb/go_daily-termdb-tables.tar.gz

^e^ftp://ftp.ebi.ac.uk/pub/databases/GO/goa/

^f^Arabidopsis, Chicken, Cow, Human, Mouse, Rat and Zebrafish

^g^http://www.ncbi.nlm.nih.gov/Taxonomy

^h^http://www.ncbi.nlm.nih.gov/sites/entrez?db=gene

## Competing interest

We declare having no competing interest.

## Author’s contributions

CB developed GO2PUB method, software and website, participated in the design and the realization of the relevance study and drafted the manuscript. CD participated in the design and the realization of the relevance study and drafted the manuscript. AB participated in the design and the realization of the relevance study and drafted the manuscript. OD participated in the design and the realization of the relevance study and drafted the manuscript. All authors read and approved the final manuscript.

## Supplementary Material

Additional file 1 This text file contents the experts selections for the query Q1. Click here for file

Additional file 2 This text file contents the experts selections for the query Q2. Click here for file

Additional file 3 This text file contents the experts selections for the query Q3. Click here for file

Additional file 4 GO2PUB results file for query Q1. Click here for file
