# Supplementary material for: GO2PUB: Querying PubMed with semantic expansion of gene ontology terms
Source: J Biomed Semantics. 2012 Sep 7;3:7. doi: 10.1186/2041-1480-3-7 (PMC3599846; doi:10.1186/2041-1480-3-7)
Supplement: Additional file 4 — GO2PUB results file for query Q1. [file 2041-1480-3-7-S4.html]

GO2PUB

 ° Your query °  
All Symbol[TIAB] OR Name[TIAB] OR Synonyms[TIAB] of genes of asked taxon(s) annotated by GO:0008610 AND ("Chickens"[MESH]) AND ("Liver"[MAJR] AND "Lipid Metabolism"[MESH])) AND ("2005"[PDat] : "2010"[PDat])
  
  
  
  
  
° Results °  
Article 1 : 20060923     2010  

#### Effects of maternal treatment of dehydroepiandrosterone (DHEA) on serum lipid profile and hepatic lipid metabolism-related gene expression in embryonic chickens

Chen J, Tang X, Zhang Y, Ma H, Zou S
  
  
Comparative biochemistry and physiology. Part B, Biochemistry & molecular biology
  
  
Abstract :

Over the last decade, much evidence emerged to suggest that alterations in maternal diets during pregnancy may irreversibly affect aspects of physiological and biochemical functions in the fetus. To explore the effects of maternal dietary treatments with dehydroepiandrosterone (DHEA) on lipid metabolism in the embryo, we investigated serum lipid profile and hepatic lipid metabolism-related gene expression in the maternal and embryonic chicken. Sixteen-week-old pullets were allocated into 3 groups (n=30), and after laying, they were provided with a commercial diet supplemented with DHEA at 0, 20 or 100mg/kg diet. Eggs were collected after DHEA treatment and incubated at 37.5 degrees C and a relative humidity of 60%. Blood and liver samples were collected from hens and embryonic chickens. DHEA treatment resulted in decreased body weight and increased relative liver weight in both maternal and embryonic chickens, while the concentrations of blood triglyceride (TG), total cholesterol (TC), low-density lipoprotein-cholesterol (LDL-C) and non-esterified fatty acid (NEFA) were significantly lower in the 20mg DHEA/kg group as compared to the control group during embryonic development. The expression of acetyl CoA carboxylase (ACC) and carnitine palmitoyl transferase I (CPTI) gene was also reduced following treatment with 20mg DHEA/kg at hatching. However, blood TC, and hepatic fatty acid synthase (FAS) and hydroxy methylglutaryl-CoA reductase (HMGR) gene expression were significantly up-regulated in the 100mg DHEA/kg group during embryonic development and hatching. Overall, the results of this study indicate that maternal dietary treatment with DHEA regulates serum lipid metabolism and hepatic gene expression.

  
  


---

  
  
Article 2 : 20049584     2010  

#### Use of comparative proteomics to identify key proteins related to hepatic lipid metabolism in broiler chickens: evidence accounting for differential fat deposition between strains

Huang J, Tang X, Ruan J, Ma H, Zou S
  
  
Lipids
  
  
Abstract :

In order to investigate differences in fat metabolism during embryonic development, a comparative proteomics strategy was employed using Arbor Acres (AA) and San Huang (SH) broiler chickens with different growth and fat deposition characteristics. These birds were floor-reared and fed identical diets, and embryonic livers were collected from AA and SH chicken embryos on days 9, 14 and 19 of incubation and hatching. Proteins were extracted and fractionated by two-dimensional electrophoresis (2-DE), Neuhoff's colloidal Coomassie Blue G-250 staining was carried out, and stained gels were scanned and analyzed using PDQuest7.3 software (Bio-Rad). In-gel trypsin digestion of the differential protein spots and matrix-assisted laser desorption/ionization-time of flight mass spectrometry (MALDI-TOF-MS) were subsequently assessed. Peptide mass fingerprinting of the differentially expressed proteins was performed using the server from MASCOT or either Prospector or ProFound, and 37 proteins were successfully identified. In the present study, embryo and liver weights showed a trend toward enhanced growth during embryonic development. Of the 37 identified differential proteins, phosphoenolpyruvate carboxykinase (PEPCK), apolipoprotein A-I (Apo A-I), fatty acid-binding protein (L-FABP) and 3-hydroxy-3-methylglutaryl-Coenzyme A synthase (HMG-CoA synthase) were up-regulated in SH chickens to a greater extent than they were in AA chickens. These observations suggest that the lipid metabolic proteins and enzymes are inherent characteristics that contribute to the apparent differences in fat deposition between the two strains.

  
  


---

  
  
Article 3 : 19427916     2009  

#### AMP-activated protein kinase and carbohydrate response element binding protein: a study of two potential regulatory factors in the hepatic lipogenic program of broiler chickens

Proszkowiec-Weglarz M, Richards MP, Humphrey BD, Rosebrough RW, McMurtry JP
  
  
Comparative biochemistry and physiology. Part B, Biochemistry & molecular biology
  
  
Abstract :

This study investigated the effects of fasting and refeeding on AMP-activated protein kinase (AMPK) and carbohydrate response element binding protein (ChREBP) mRNA, protein and activity levels; as well as the expression of lipogenic genes involved in regulating lipid synthesis in broiler chicken (Gallus gallus) liver. Fasting for 24 or 48 h produced significant declines in plasma glucose (at 24 h), insulin and thyroid hormone (T3) levels that were accompanied by changes in mRNA expression levels of hepatic lipogenic genes. The mRNA levels of malic enzyme (ME), ATP-citrate lyase (ACL), acetyl-CoA carboxylase alpha (ACCalpha), fatty acid synthase (FAS), stearoyl-CoA desaturase-1 (SCD-1) and thyroid hormone responsive Spot 14 (Spot 14) declined in response to fasting. Refeeding for 24 h increased mRNA levels for each of these genes, characterized by a significant increase ('overshoot') above fed control values. No change in mRNA expression of the two AMPK alpha subunit genes was observed in response to fasting or refeeding. In contrast, ChREBP and sterol regulatory element binding protein-1 (SREBP-1) mRNA levels decreased during fasting and increased with refeeding. Phosphorylation of AMPK alpha subunits increased modestly after a 48 h fast. However, there was no corresponding change in the phosphorylation of ACC, a major downstream target of AMPK. Protein level and DNA-binding activity of ChREBP increased during fasting and declined upon refeeding as measured in whole liver tissue extracts. In general, evidence was found for coordinate transcriptional regulation of lipogenic program genes in broiler chicken liver, but specific regulatory roles for AMPK and ChREBP in that process remain to be further characterized.

  
  


---

  
  
Article 4 : 19393339     2009  

#### Increased de novo lipogenesis in liver contributes to the augmented fat deposition in dexamethasone exposed broiler chickens (Gallus gallus domesticus)

Cai Y, Song Z, Zhang X, Wang X, Jiao H, Lin H
  
  
Comparative biochemistry and physiology. Toxicology & pharmacology : CBP
  
  
Abstract :

Effect of dexamethasone (DEX, a synthetic glucocorticoid) on lipid metabolism in broiler chickens (Gallus gallus domesticus) was investigated. Male Arbor Acres chickens (1 wk old, n=30) were injected with DEX or saline for 1 wk, and a pair-fed group was included. DEX administration resulted in enhanced lipid deposition in adipose tissues. Plasma insulin increased about 3.3 fold in DEX injected chickens as against the control and hepatic triglyceride was higher as compared with the pair-fed chickens. In DEX injected chickens, the hepatic activities of malic enzyme (ME) and fatty acid synthetase (FAS) were significantly increased, while the mRNA levels of acetyl CoA carboxylase (ACC), ME, and FAS were significantly up-regulated, compared with the control. Although the mRNA levels of lipoprotein lipase (LPL), peroxisome proliferator-activated receptor-gamma (PPARgamma) and adipose triglyceride lipase (ATGL) genes in adipose tissue were not affected by DEX injection, ME activity and mRNA levels in abdominal fat pad of chickens treated with DEX are higher than those of control chickens. The results indicated that the increased hepatic de novo lipogenesis and in turn, the increased circulating lipid flux contributes to the augmented fat deposition in adipose tissues and liver in DEX-challenged chickens. The results suggest that glucocorticoids together with the induced hyperinsulinemia should be responsible for the up-regulated hepatic lipogenesis.

  
  


---

  
  
Article 5 : 19383549     2009  

#### The effect of feed restriction on expression of hepatic lipogenic genes in broiler chickens and the function of SREBP1

Wang PH, Ko YH, Chin HJ, Hsu C, Ding ST, Chen CY
  
  
Comparative biochemistry and physiology. Part B, Biochemistry & molecular biology
  
  
Abstract :

To study the role of sterol regulatory element-binding proteins (SREBP) in lipogenesis and cholesterol synthesis in the chicken, two experiments were carried out. In the first study, seven-week-old broilers (n=16) were allocated into 2 groups, fasted for 24 h or refed for 5 h after a 24 h fasting. The mRNA concentrations for SREBPs and other lipogenic genes in the liver were determined by quantitative real time PCR. The hepatic mRNA relative abundance of lipogenic genes and genes involved in cholesterol synthesis were significantly greater (p<0.001) in the refed broilers. Similar results were demonstrated with Northern analysis. The data suggest that in the liver of fasted broilers, genes associated with lipogenesis and cholesterol biosynthesis were inhibited. Indeed, the mRNA concentrations for fatty acid synthase (FAS), malic enzyme, and stearoyl coenzyme A desaturase were almost undetectable after the 24 h fasting. The data also demonstrated that the expression of lipogenic genes coordinate well as a group during the refeeding period. Second, three small interfering RNA (siRNA) oligonucleotides against SREBP1 were designed to be used in transfecting a chicken hepatocarcinoma cell line LMH. One of the three siRNAs effectively reduced SREBP1 mRNA concentration (p<0.01). The acetyl coenzyme A carboxylase(alpha) (ACC(alpha)) mRNA was also significantly reduced by the SREBP1 siRNA treatment, suggesting that SREBP1 can upregulate the expression of this lipogenic gene. This siRNA, however, did not affect the mRNA for FAS. Taken together, the RNA interference study showed that SREBP1 has the ability to regulate the expression of ACC(alpha). This study has helped us understand more about the function of SREBP1 and the physiology of the broiler chickens.

  
  


---

  
  
Article 6 : 19091074     2008  

#### Transcriptome profiling of the feeding-to-fasting transition in chicken liver

Désert C, Duclos MJ, Blavy P, Lecerf F, Moreews F, Klopp C, Aubry M, Herault F, Le Roy P, Berri C, Douaire M, Diot C, Lagarrigue S
  
  
BMC genomics
  
  
Abstract :

Starvation triggers a complex array of adaptative metabolic responses including energy-metabolic responses, a process which must imply tissue specific alterations in gene expression and in which the liver plays a central role. The present study aimed to describe the evolution of global gene expression profiles in liver of 4-week-old male chickens during a 48 h fasting period using a chicken 20 K oligoarray.

  
  


---

  
  
Article 7 : 17574488     2007  

#### Effects of in ovo administration of DHEA on lipid metabolism and hepatic lipogenetic genes expression in broiler chickens during embryonic development

Zhao S, Ma H, Zou S, Chen W
  
  
Lipids
  
  
Abstract :

In order to study the mechanism of DHEA (Dehydroepiandrosterone) in reducing fat in broiler chickens during embryonic development, fertilized eggs were administrated with DHEA before incubation and its effect on lipid metabolism and expression of hepatic lipogenetic genes was investigated. The mRNA levels of acetyl CoA carboxylase (ACC), fatty acid synthase (FAS), malic enzyme (ME), apolipoprotein B100 (apoB100) and sterol regulator element binding protein-1c (SREBP-1c) were determined using real time quantitative PCR. Samples of livers were collected from the chickens on days 9, 14, and 19 of embryonic development as well as at hatching. Blood samples were extracted on days 14, 19 of incubation and at hatching. The results showed that DHEA decreased the concentration of triacyglycerol in the blood and the content in liver, and the mRNA levels of ACC, FAS, ME, SREBP-1c and apoB. This suggested that DHEA decreased the expression of hepatic lipogenetic genes and suppressed triglycerols transport, by which it reduced the deposition of fat in adipose tissue in broiler chickens during embryonic development and hatching.

  
  


---

  
  
Article 8 : 17626819     2007  

#### An examination of the role of feeding regimens in regulating metabolism during the broiler breeder grower period. 1. Hepatic lipid metabolism

de Beer M, Rosebrough RW, Russell BA, Poch SM, Richards MP, Coon CN
  
  
Poultry science
  
  
Abstract :

A trial was conducted to determine the effects of feeding regimens on hepatic lipid metabolism in 16-wk-old broiler breeder pullets. A flock of 350 Cobb 500 breeder pullets was divided into 2 at 4 wk of age and fed either every day (ED) or skip-a-day (SKIP) from 4 to 16 wk of age. Total feed intake did not differ between the 2 groups. At 112 d, 52 randomly selected ED-fed pullets, and 76 SKIP-fed pullets were individually caged and fed a 74-g (ED) or 148-g (SKIP) meal. Four pullets from each group were killed at intervals after feeding and livers were collected, weighed, and snap-frozen for determination of lipogenic gene expression. Total RNA was isolated from livers using Trizol reagent and then quantitatively measured by noting the optical density 260:280 ratio and qualitatively measured by gel electrophoresis. The expression of certain regulatory genes in metabolism [acetyl coenzyme A carboxylase; fatty acid synthase; malic enzyme (MAE); isocitrate dehydrogenase (ICDH); and aspartate aminotransferase (AAT)] were determined by real-time reverse-transcription PCR. Remaining liver portions were analyzed for enzyme activity of MAE, ICDH, and AAT as well as glycogen and lipid contents. Liver weight was higher in SKIP than in ED birds. Feeding caused dramatic increases in liver weight, glycogen, and lipids of SKIP birds. Expression of acetyl coenzyme A carboxylase, FAS, and MAE genes were increased in SKIP birds 12 and 24 h after feeding, with the increases in MAE expression from 0 to 24 h after feeding being of the greatest magnitude. In contrast, SKIP decreased ICDH and AAT gene expression, which parallels findings noted in fasting-refeeding experiments conducted with much younger birds. Skip-a-day feeding resulted in far greater changes in gene expression compared with ED, which was indicative of the inconsistent supply of nutrients in such regimens. Enzyme activity of MAE, ICDH, and AAT was reflective of noted changes in gene expression. In summary, the feeding regimen greatly affected hepatic gene expression in breeder pullets.

  
  


---

  
  
Article 9 : 15941644     2005  

#### Potential role of leptin in increase of fatty acid synthase gene expression in chicken liver

Dridi S, Buyse J, Decuypere E, Taouis M
  
  
Domestic animal endocrinology
  
  
Abstract :

Leptin is reported to have direct effects on lipogenesis in peripheral tissues that are independent of its central effects on food intake and body weight. These experiments have been mainly carried out in rodents (different models of obesity) in which lipogenesis occurs in both adipose tissue and liver. Such effects are unknown in birds in which lipogenesis occurs essentially in the liver. In this study, leptin gene expression and circulating leptin levels were examined in two chicken lines, selected for high (FL) or low (LL) abdominal fat pad size, at different nutritional states (fasted and fed state). In addition, effects of recombinant chicken leptin on liver metabolism were investigated. Hepatic leptin and fatty acid synthase (FAS) gene expression and plasma leptin levels were significantly higher in FL than in LL chickens (P < 0.05). In both lines, fasting significantly reduced hepatic leptin and FAS mRNA levels (P < 0.05). Continuous administration of recombinant chicken leptin (8 microg/kg/h) during 6 h significantly inhibited food intake (51%) and increased leptinemia (23-fold) compared to untreated group. Despite the decrease of food intake, leptin significantly induced the expression of FAS in chicken liver. These changes were accompanied by a significant down-regulation of leptin receptor gene expression, however SREBP-1, the main transcription factor of lipogenic genes, remained unchanged. This result suggests a local potential role of leptin in the regulation of avian hepatic lipogenesis, and explain, at least partly, the metabolic changes evolved during the divergent selection of FL and LL chickens.

  
  
  
  
° Keywords searched in [MeSH] °  
All Symbols[TIAB] OR Names[TIAB] OR Synonyms[TIAB] of genes of asked taxon(s) annotated by GO:0008610 AND ("Chickens"[MESH]) AND ("Liver"[MESH] AND "Lipid Metabolism"[MESH])) AND ("2005"[PDat] : "2010"[PDat])   
  
  
  
  
° Results °  
Article 10 : 20138241     2010  

#### Dexamethasone facilitates lipid accumulation and mild feed restriction improves fatty acids oxidation in skeletal muscle of broiler chicks (Gallus gallus domesticus)

Wang X, Lin H, Song Z, Jiao H
  
  
Comparative biochemistry and physiology. Toxicology & pharmacology : CBP
  
  
Abstract :

Effects of dexamethasone (DEX) and mild feed restriction on the uptake and utilization of fatty acids in skeletal muscle of broiler chicks (Gallus gallus domesticus) were investigated. Male Arbor Acres chicks (7-days old, n=30) were injected with DEX or saline for 3days, and a feed restriction group was included. DEX enhanced circulating very low density lipoprotein (VLDL) level and the lipid accumulation in both adipose and skeletal muscle tissues. Compared with the control, liver-carnitine palmitoyltransferase 1 (L-CPT1) and AMP-activated protein kinase (AMPK) alpha2 mRNA level of M. biceps femoris (BF) were down-regulated significantly by DEX, while mRNA expression of lipoprotein lipase (LPL), fatty acid transport protein 1 (FATP1), heart-fatty acid binding protein (H-FABP), long-chain acyl-CoA dehydrogenase (LCAD), activities of LPL and AMPK in both skeletal muscles were not obviously affected. Feed restriction increased the mRNA expression of LPL, L-CPT1 and LCAD of M. pectoralis major (PM), and FATP1, H-FABP, L-CPT1 and LCAD of BF. In conclusion, DEX retards the growth of body mass but facilitates lipid accumulation in both adipose and skeletal muscle tissues. In contrast to the favorable effect of mild feed restriction, DEX did not alter the uptake of fatty acids in the skeletal muscle. The result suggests that DEX may promote intramyocellular lipid accumulation by suppressed fatty acid oxidation while mild feed restriction improved fatty acid oxidation in skeletal muscle, especially in red muscle. Glucocorticoids (GCs) regulated muscle fatty acid metabolism in a different way from energy deficit caused by mild feed restriction.

  
  


---

  
  
Article 11 : 20003570     2010  

#### Effect of dietary sodium phytate and microbial phytase on the lipase activity and lipid metabolism of broiler chickens

Liu N, Ru Y, Wang J, Xu T
  
  
The British journal of nutrition
  
  
Abstract :

The objective of the present study was to investigate the effect of dietary phytate and microbial phytase on the lipase activity, lipid metabolism and mRNA expressions of fatty acid synthase (FASN) and leptin in broiler chickens. The study was conducted as a 2 x 3 factorial arrangement of treatments with phytate phosphorus at 0.20 and 0.40 % (added as the sodium phytate) and supplemental microbial phytase at 0, 500, or 1000 phytase units/kg. The results showed that phytase improved (P < 0.05) the growth performance and ileal digestibility of nutrients of broilers, but phytate had no effect (P>0.05) on these parameters, except the decrease (P < 0.01) in the digestibility of Ca. Phytate decreased (P < 0.05) the lipase activity, serum total cholesterol (T-CHO) and hepatic TAG, and elevated (P < 0.01) serum NEFA and HDL cholesterol. Phytase decreased (P < 0.05) serum NEFA, but increased (P < 0.01) serum T-CHO and hepatic TAG. Phytate and phytase also influenced (P < 0.01) the mRNA expressions of leptin in the liver. There were significant (P < 0.05) interactions of phytate and phytase on the concentrations of serum TAG and LDL cholesterol, hepatic NEFA and T-CHO, and the mRNA expressions of FASN. The results suggest that phytate and phytase can affect lipase activity and lipid metabolism of broiler chickens.

  
  


---

  
  
Article 12 : 19231861     2009  

#### Effect of an oil byproduct from conjugated linoleic acid (CLA) purification on CLA accumulation and lipogenic gene expression in broilers

Kim JH, Jeong WS, Kim IH, Kim HJ, Kim SH, Kang GH, Lee HG, Yoon HG, Ham HJ, Kim YJ
  
  
Journal of agricultural and food chemistry
  
  
Abstract :

A previous study showed that supplementing broilers with an oil byproduct obtained during the purification process of conjugated linoleic acid (CLA) from safflower oil could result in CLA-enriched egg yolks more efficiently than feeding purified CLA (free fatty acid form). On this basis, this study evaluated whether dietary CLA byproduct (CBP) supplementation would enhance CLA accumulation in broiler muscle and its lipogenic mRNA expression in the liver. A total of 456 1-day-old male broiler chicks were randomly assigned to four groups, each of which was given one of the following 2% dietary supplements for 4 weeks: soybean oil (control), safflower oil (SAF), purified CLA, and CBP. During the feeding trial, little alteration in broiler performance was observed among the test groups. CLA accumulation efficiency in the breast muscle did not differ significantly between the CLA- and CBP-fed groups after feeding of the test diet for 3 weeks. CLA supplementation also induced lipogenesis in the livers of the broilers, and it significantly increased the relative mRNA levels of sterol regulatory element binding protein 1 (SREBP1), as well as its target genes: fatty acid synthase (FAS) and acetyl coenzyme A carboxylase (ACC) (p < 0.05). However, in the CBP-fed group, SREBP1 and ACC mRNA levels were not significantly different from the controls (p > 0.05). These results suggest that CBP could be an efficient dietary source that promotes CLA accumulation in broiler muscle without inducing lipogenesis in the liver or compromising performance and meat quality in the birds.

  
  


---

  
  
Article 13 : 18313342     2008  

#### Short term changes in the expression of lipogenic genes in broilers (Gallus gallus)

Rosebrough RW, Russell BA, Richards MP
  
  
Comparative biochemistry and physiology. Part A, Molecular & integrative physiology
  
  
Abstract :

The purpose of these experiments were to determine possible relationships between certain indices of lipid metabolism and specific gene expression in chickens fed graded levels of dietary crude protein. Male, broiler chickens growing from 7 to 28 days of age were fed diets containing 12 or 30% protein ad libitum. Both groups were then switched on day 28 to the diets containing the opposite level of protein. Birds were killed on day 28 (basal values prior to the switch) and at 12, 18 and 24 h post switch. Measurements taken included in vitro lipogenesis, malic enzyme activity the expression of the genes for malic enzyme, fatty acid synthase and acetyl coenzyme carboxylase. In vitro lipogenesis and malic enzyme activity were inversely related to dietary protein levels (12 to 30%) and to acute changes from 12 to 30%. Malic enzyme, fatty acid synthase and acetyl coenzyme A carboxylase genes were constant over a dietary protein range of 12 to 21% as in previous experiments, but decreased by feeding a 30% protein diet in the present experiments (acute or chronic feeding). Results of the present study demonstrate a continued role for protein in the regulation of broiler metabolism. Metabolic regulation at the gene level only occurs when feeding very high levels of dietary protein.

  
  


---

  
  
Article 14 : 17359447     2007  

#### Hepatic lipogenesis in broiler chickens with different fat deposition during embryonic development

Zhao S, Ma H, Zou S, Chen W, Zhao R
  
  
Journal of veterinary medicine. A, Physiology, pathology, clinical medicine
  
  
Abstract :

In order to identify the genes involved in the fatness variability, we studied the expression of several genes implicated in the hepatic lipid metabolism of broiler chickens with different fat deposition patterns during embryonic development. The mRNA expression of acetyl CoA carboxylase (ACC), fatty acid synthase (FAS), malic enzyme (ME) and apolipoprotein B100 (apoB100) genes were determined using reverse transcriptase-polymerase chain reaction (RT-PCR). Samples of livers were collected from Arbor Acres (AA) and Sanhuang (SH) chickens on day 9, 14 and 19 of embryonic development as well as at hatching. This study showed that hepatic triglyceride (TG) level was found to increase suddenly during day 14 of embryonic development, to gradually increase thereafter, and to remain relatively constant at hatching. FAS gene expression in AA and SH broilers occurred prior to hatching and at hatching. The gene was expressed more in the former breed. ACC gene expression was observed beginning at the earlier development stage of days 9. No breed difference was observed in ME and apoB gene expression. This study indicated that the expression of lipogenic enzyme genes of the liver in broiler chickens exhibited scheduling during embryogenesis. The ACC gene started to express earlier than the FAS gene during embryonic development. This suggested that embryonic liver synthesized fatty acid, and breed difference was noticed prior to hatching.

  
  


---

  
  
Article 15 : 17495091     2007  

#### Effects of clofibrate treatment in laying hens

König B, Kluge H, Haase K, Brandsch C, Stangl GI, Eder K
  
  
Poultry science
  
  
Abstract :

Expression of peroxisome proliferator-activated receptor-alpha (PPARalpha) has been shown in liver of chicks, but effects of its activation have not yet been investigated. In this study, laying hens were treated with clofibrate, a synthetic PPARalpha agonist, to investigate the effects of PPARalpha activation on liver lipid metabolism. Hens receiving a diet containing 5 g of clofibrate/kg had a lower food intake and higher liver mRNA concentrations of typical PPARalpha target genes (carnitine palmitoyltransferase 1A, acyl-coenzyme A oxidase, bifunctional enzyme, lipoprotein lipase) involved in hepatic mitochondrial and peroxisomal beta-oxidation and plasma triglyceride clearance than control hens that received the same diet without clofibrate (P<0.05). Hens treated with clofibrate also had lower mRNA concentrations of fatty acid synthase, 3-hydroxy-3-methylglutaryl-coenzyme A reductase, and low-density lipoprotein receptor, proteins involved in fatty acid biosynthesis and cholesterol biosynthesis and uptake, than hens fed the control diet (P<0.05). These changes in clofibrate-treated hens were accompanied by reduced liver triglyceride concentrations, strongly diminished very low density triglyceride and cholesterol concentrations (P<0.05), a disturbed maturation of egg follicles, a complete stop of egg production, and a markedly reduced plasma 17-beta-estradiol concentration (P<0.05). In conclusion, it is shown that clofibrate has complex effects on hepatic lipid metabolism in laying hens that mimic PPARalpha activation in mammals, affect maturation of egg follicles, and lead to a stop of egg production. Because clofibrate treatment strongly reduced food intake in the hens, some of these effects (i.e., egg production) may have been due to a low energy and nutrient intake.

  
  


---

  
  
Article 16 : 16413806     2006  

#### Effects of atorvastatin on lipid metabolism in normolipidemic and hereditary hyperlipidemic, non-laying hens

Elkin RG, Zhong Y, Donkin SS, Hengstschläger-Ottnad E, Schneider WJ
  
  
Comparative biochemistry and physiology. Part B, Biochemistry & molecular biology
  
  
Abstract :

As a result of a hereditable point mutation in the oocyte very low density lipoprotein (VLDL) receptor, sexually mature restricted ovulator (RO) female chickens (Gallus gallus), first described as a non-laying strain, exhibit endogenous hyperlipidemia and develop atherosclerotic lesions. In a 20-day study, RO hens and their normolipidemic (NL) siblings were fed either a control diet, or the control diet supplemented with 0.06% atorvastatin (AT), a potent 3-hydroxy-3-methylglutaryl-coenzyme A reductase (HMGR) inhibitor. Compared to NL hens, RO birds exhibited greatly elevated baseline plasma total cholesterol (CHOL) and triglyceride (TG) concentrations (1.56 vs. 4.55 g/l and 30.7 vs. 138.4 g/l, respectively). AT attenuated plasma CHOL and TG concentrations by 60.3% and 70.1%, respectively, in NL hens and by 45.1% and 34.3%, respectively, in RO hens. Messenger RNA levels of several key genes involved in hepatic VLDL assembly were suppressed in RO vs. NL hens, but were unaffected by AT. In contrast, AT elevated liver HMGR mRNA levels in NL and RO birds, but only NL hens exhibited an AT-associated increase in hepatic HMGR immunoreactive protein levels. Down-regulation of HMGR gene expression due to higher baseline levels of circulating CHOL may explain why RO birds responded less robustly than NL hens to AT administration.

  
  
  
  
° Keywords linked by "OR" connector °  
All Symbols[TIAB] OR Names[TIAB] OR Synonyms[TIAB] of genes of asked taxon(s) annotated by GO:0008610 AND ("Chickens"[MAJR]) AND("Liver"[MAJR] OR "Lipid Metabolism"[MAJR])) AND ("2005"[PDat] : "2010"[PDat])   
  
  
  
  
° Results °  
Article 17 : 19903961     2009  

#### Effects of turmeric (Curcuma longa) on the expression of hepatic genes associated with biotransformation, antioxidant, and immune systems in broiler chicks fed aflatoxin

Yarru LP, Settivari RS, Gowda NK, Antoniou E, Ledoux DR, Rottinghaus GE
  
  
Poultry science
  
  
Abstract :

The objective of the present study was to evaluate the efficacy of curcumin, an antioxidant found in turmeric (Curcuma longa) powder (TMP), to ameliorate changes in gene expression in the livers of broiler chicks fed aflatoxin B(1) (AFB(1)). Four pen replicates of 5 chicks each were assigned to each of 4 dietary treatments, which included the following: A) basal diet containing no AFB(1) or TMP (control), B) basal diet supplemented with TMP (0.5%) that supplied 74 mg/kg of curcumin, C) basal diet supplemented with 1.0 mg of AFB(1)/kg of diet, and D) basal diet supplemented with TMP that supplied 74 mg/kg of curcumin and 1.0 mg of AFB(1)/kg of diet. Aflatoxin reduced (P < 0.05) feed intake and BW gain and increased (P < 0.05) relative liver weight. Addition of TMP to the AFB(1) diet ameliorated (P < 0.05) the negative effects of AFB(1) on growth performance and liver weight. At the end of the 3-wk treatment period, livers were collected (6 per treatment) to evaluate changes in the expression of genes involved in antioxidant function [catalase (CAT), superoxide dismutase (SOD), glutathione peroxidase (GPx), glutathione S-transferase (GST)], biotransformation [epoxide hydrolase (EH), cytochrome P450 1A1 and 2H1 (CYP1A1 and CYP2H1)], and the immune system [interleukins 6 and 2 (IL-6 and IL-2)]. Changes in gene expression were determined using the quantitative real-time PCR technique. There was no statistical difference in gene expression among the 4 treatment groups for CAT and IL-2 genes. Decreased expression of SOD, GST, and EH genes due to AFB(1) was alleviated by inclusion of TMP in the diet. Increased expression of IL-6, CYP1A1 and CYP2H1 genes due to AFB(1) was also alleviated by TMP. The current study demonstrates partial protective effects of TMP on changes in expression of antioxidant, biotransformation, and immune system genes in livers of chicks fed AFB(1). Practical application of the research is supplementation of TMP in diets to prevent or reduce the effects of aflatoxin in chicks fed aflatoxin-contaminated diets.

  
  


---

  
  
Article 18 : 19373719     2009  

#### Influence of dietary vitamin E supplementation on meat quality traits and gene expression related to lipid metabolism in the Beijing-you chicken

Li WJ, Zhao GP, Chen JL, Zheng MQ, Wen J
  
  
British poultry science
  
  
Abstract :

1. The effects of dietary vitamin E (DL-alpha-tocopheryl acetate) on carcase and meat quality, oxidative stability, fatty acid composition of muscle lipids, and gene expression related to lipid metabolism were studied in Beijing-you chickens. 2. A total of 360 female birds were distributed among 6 treatments, containing 6 replicates, each of 10 birds. The feed for each treatment was supplemented with vitamin E (0, 10, 50, 100, 150, or 200 mg/kg feed). At 120 d, 30 birds from each treatment were slaughtered to examine the effect of dietary vitamin E supplementation on evaluated traits. 3. The results showed that supplemental vitamin E in diet significantly increased alpha-tocopherol contents of breast and thigh muscles, reduced the drip loss and improved tenderness but did not influence carcase yield, meat colour or pH value 24 h after slaughter. 4. Thiobarbituric acid reactive substance (TBARS) values decreased with increase in dietary vitamin E, and the addition of 100 mg/kg or more vitamin E had a beneficial effect on oxidative stability as indicated by TBARS values during storage up to 7 d. 5. Dietary vitamin E supplementation significantly altered fatty acid composition of breast muscle. Supplementing with 200 mg/kg vitamin E led to lower saturated fatty acids and greater polyunsaturated fatty acids proportions in breast muscle than control and 10 mg/kg vitamin E treatments. 6. Vitamin E supplementation significantly inhibited expression of the cytosolic phospholipase A(2) gene (cPLA(2)) in breast muscle, while enhancing that of the peroxisome proliterator-activated receptor beta (PPAP-beta) and heart fatty acid binding protein genes (H-FABP). The results indicate that dietary supplementation with vitamin E increased lipid stability in muscle and improved meat quality and fatty acid composition, probably by its influence on the expression of genes related to lipid metabolism.

  
  


---

  
  
Article 19 : 19151351     2009  

#### Toxicological and gene expression analysis of the impact of aflatoxin B1 on hepatic function of male broiler chicks

Yarru LP, Settivari RS, Antoniou E, Ledoux DR, Rottinghaus GE
  
  
Poultry science
  
  
Abstract :

The objective of this study was to determine the effects of dietary aflatoxin B1 (AFB1) on hepatic gene expression in male broiler chicks. Seventy-five 1-d-old male broiler chicks were assigned to 3 dietary treatments (5 replicates of 5 chicks each) from hatch to d 21. The diets contained 0, 1 and 2 mg of AFB1/kg of feed. Aflatoxin B1 reduced (P<0.05) feed intake, BW gain, serum total proteins, and serum Ca and P, but increased (P<0.01) liver weights in a dose-dependent manner. Microarray analysis was used to identify shifts in genetic expression associated with the affected physiological processes in chicks fed 0 and 2 mg of AFB1/kg of feed to identify potential targets for pharmacological/toxicological intervention. A loop design was used for microarray experiments with 3 technical and 4 biological replicates per treatment group. Ribonucleic acid was extracted from liver tissue, and its quality was determined using gel electrophoresis and spectrophotometry. High-quality RNA was purified from DNA contamination, reverse transcribed, and hybridized to an oligonucleotide microarray chip. Microarray data were analyzed using a 2-step ANOVA model and validated by quantitative real-time PCR of selected genes. Genes with false discovery rates less than 13% and fold change greater than 1.4 were considered differentially expressed. Compared with controls (0 mg of AFB1/kg), various genes associated with energy production and fatty acid metabolism (carnitine palmitoyl transferase), growth and development (insulin-like growth factor 1), antioxidant protection (glutathione S transferase), detoxification (epoxide hydrolase), coagulation (coagulation factors IX and X), and immune protection (interleukins) were downregulated, whereas genes associated with cell proliferation (ornithine decarboxylase) were upregulated in birds fed 2 mg of AFB1/kg. This study demonstrates that AFB1 exposure at a concentration of 2 mg/kg results in physiological responses associated with altered gene expression in chick livers.

  
  


---

  
  
Article 20 : 18038926     2007  

#### Growth characteristics of the Ross 708 broiler chicken

Rosebrough RW, Mitchell AD
  
  
Growth, development, and aging : GDA
  
  
Abstract :

A growth trial was conducted with the Ross 708 broiler chicken to corroborate the relationships between changes in the growth curve (7 to 35 days) and in vitro metabolic parameters. These in vitro parameters also included estimates of the expression of certain genes regulating proteins implicated with regulation of lipogenesis. Birds were fed diets containing 24% protein from 0 to 14 days of age, 21% from 14 to 26 days of age and 18% protein until 35 days of age. Birds were selected and killed at ages corresponding to protein changes. Dual X-ray absorptiometry (DXA) was used to approximate body composition of birds at day 35. The switch from the starter protein level of 24% crude protein to the only slightly lower protein grower diet (21% crude protein) increased both in vitro lipogenesis and malic enzyme activity. A similar observation was noted when the birds were switched to the 18% crude protein finisher diet. These same switches also elicited initial increases in malic enzyme, fatty acids synthase and acetyl CoA carboxylase gene expression that were not sustained following adaptation to the dietary change. Data also show that DXA can be used to estimate body composition of this type of bird.

  
  


---

  
  
Article 21 : 17704960     2007  

#### Effects of dehydroepiandrosterone (DHEA) on hepatic lipid metabolism parameters and lipogenic gene mRNA expression in broiler chickens

Tang X, Ma H, Zou S, Chen W
  
  
Lipids
  
  
Abstract :

The aim of the present study was to identify the effects of dehydroepiandrosterone (DHEA) on hepatic lipid metabolism parameters and lipogenic gene mRNA expression in broiler chickens. A total of 72 1-day-old broiler chicks received a common basal diet with DHEA added at either 0 (control), 5 or 20 mg/kg feed. In the present study, the hepatic triglyceride (TG) concentration was significantly lower in male and female broilers that had bed administered DHEA than in control birds. In contrast, DHEA administration caused a marked rise in the hepatic non-esterified fatty acid (NEFA) concentration in both male and female broilers and also increased lipase (HL) activity in male broilers, while in female birds, no significant differences were observed in HL activity. The expression of peroxisome proliferators-activated receptor alpha (PPARalpha) and carnitine palmitoyl transferase I (CPTI) mRNA was decidedly enhanced following treatment with DHEA, and a similar tendency was also observed in the expression of acyl-Coenzyme A oxidase 1 (ACOX1). However, no significant differences were observed in the expression of either sterol regulatory element binding protein-1c (SREBP-1c) or acetyl CoA carboxylase (ACC) mRNA, except for a decline in the expression of ACC in females treated with 5 mg DHEA/kg. Numerous peroxisomes without a core and an increased number of peroxisomes were evident during morphological observations of broiler livers, in animals that had been treated with DHEA. Overall, the results of the present study indicated that DHEA accelerated lipid catabolism by direct regulation of hepatic lipid metabolism and by induction of relevant gene expression.

  
  


---

  
  
Article 22 : 17289415     2007  

#### Expression of lipogenic enzymes in chickens

Rosebrough RW, Russell BA, Poch SM, Richards MP
  
  
Comparative biochemistry and physiology. Part A, Molecular & integrative physiology
  
  
Abstract :

Hubbard x Hubbard chickens (Gallus gallus) growing from 7 to 28 days of age were fed 12 or 30% protein diets and then switched to the diets containing the opposite level of protein. Birds were killed on days 28, 29, 30 and 31. Measurements taken included in vitro lipogenesis (IVL), malic enzyme (ME), isocitrate dehydrogenase (ICD) and aspartate aminotransferase (AAT) activities and the expression of the genes for ME, fatty acid synthase (FAS) and acetyl coenzyme carboxylase (ACC). Gene expression was determined with a combined RT-PCR using SYBR green as a fluorescent probe monitored in a real time mode. IVL and ME activity were inversely related to dietary protein levels (12 to 30%) and to acute changes in either level. In contrast, both ICD and AAT activities were increased by any increase in dietary protein. Lipogenic gene expression was inversely related to protein level, whether fed on an acute or chronic basis. It appears that real time RT-PCR is an acceptable method of estimating gene expression in birds. In addition, further work will focus on primer sizes that might further optimize RT-PCR as an instrument for studying the regulation of avian lipid metabolism. Results of the present study demonstrate a continued role for protein in the regulation of broiler metabolism. However, it should be pointed out that metabolic regulation at the gene level only occurs when feeding very high levels of dietary protein.

  
  


---

  
  
Article 23 : 17032819     2006  

#### Tissue expression and association with fatness traits of liver fatty acid-binding protein gene in chicken

Wang Q, Li H, Li N, Leng L, Wang Y
  
  
Poultry science
  
  
Abstract :

Fatty acid-binding proteins belong to a superfamily of lipid-binding proteins that exhibit a high affinity for long-chain fatty acids and appear to function in metabolism and intracellular transportation of lipids. The current study was designed to investigate expression characterization and association with growth and composition traits of the liver fatty acid-binding protein (L-FABP) gene in the chicken. Northern blot analysis indicated that the gene, similar to the mammal L-FABP gene, was expressed only in liver and intestinal tissues. The mRNA levels of the chicken L-FABP gene in liver and intestine had significant differences between the broilers and Baier layers. The China Agricultural University F(2) population was used in the present study. Body weight and body composition traits were measured in the populations. Primers for the coding region and 5' upstream region of the L-FABP gene were designed according to chicken genomic and cDNA sequence. Polymorphisms were detected by DNA sequencing, and the PCR single-strand conformation polymorphisms method was developed to genotype the F(2) population. The results indicated that the L-FABP gene polymorphisms were associated with abdominal fat weight and percentage of abdominal fat, and the L-FABP gene could be a candidate locus or linked to a major gene(s) that affects fatness traits in the chicken. The results of the current study provided basic molecular information for studying the role of the L-FABP gene in the regulation of lipid metabolism in avian species.

  
  


---

  
  
Article 24 : 16513294     2006  

#### Microarray analysis of differential gene expression in the liver of lean and fat chickens

Bourneuf E, Hérault F, Chicault C, Carré W, Assaf S, Monnier A, Mottier S, Lagarrigue S, Douaire M, Mosser J, Diot C
  
  
Gene
  
  
Abstract :

Excessive adiposity has become a major drawback in meat-type chicken production. However, few studies were conducted to analyze the liver expression of genes involved in pathways and mechanisms leading to adiposity. A previous study performed by differential display on RNAs extracted from chicken livers from lean and fat lines allowed us to isolate cDNA products of genes with putative differential expression. In this study, a cDNA microarray resource was developed from these products together with cDNAs from genes involved in or related to lipid metabolism. This resource was used to analyze gene expression in the liver from lean and fat chickens. Some genes were found with a difference in expression between lean and fat animals and/or correlated to adipose tissue weight. Cytochrome P450 2C45, thought to play a role in biotransformation of steroids and poly-unsaturated fatty acids, was more expressed in lean chickens whereas fatty acid synthase, stearoyl-CoA desaturase, sterol response element binding factor 1 and hepatocyte nuclear factor 4, respectively involved in lipogenesis and its regulation, were more expressed in fat chickens. These results indicate that mechanisms involved in the expression and regulation of lipogenic genes could play a key role in fatness ontogenesis in chickens from lean and fat lines.

  
  
  
  
